# Clinical features and prognosis of the adult patients positive for both N-methyl-d-aspartate receptor and myelin oligodendrocyte glycoprotein antibodies

**DOI:** 10.3389/fimmu.2025.1664666

**Published:** 2025-10-16

**Authors:** Yifang Ma, Binting Liu, Xiaojiao Ci, Jie Lu

**Affiliations:** Department of Neurology, The Affiliated Brain Hospital of Nanjing Medical University, Nanjing, China

**Keywords:** N-methyl-D-aspartate receptor (NMDAr), myelin oligodendrocyte glycoprotein (MOG), adult, overlapping syndrome, MNOS, clinical features

## Abstract

**Introduction:**

The coexistence of the MOG antibody (MOG-ab) and NMDAR antibody (NMDAR-ab), which is known as the MOG-ab and NMDAR-ab overlapping syndrome (MNOS), is the most common in overlapping syndromes of neural autoantibodies. The study aimed to analyze the clinical features and outcomes in adult patients suffering from MNOS and investigate the mechanisms of the disorder.

**Methods:**

Adult patients suffering from MNOS admitted to the Affiliated Brain Hospital of Nanjing Medical University from 2020 to 2024 was systematically reviewed. The clinical symptoms, laboratory data, imaging results, treatments, and prognoses were evaluated. Moreover, the clinical data of MNOS patients were compared with those of anti-NMDAR encephalitis (anti-NMDARE) patients and MOG antibody-associated disorders (MOGAD) patients, respectively.

**Results:**

The study identified 23 adult patients of MNOS. The study cohort comprised 20 males (86.96%) and 3 females (13.04%), with a mean age of onset of 36.17 ± 11.21 years. Prodromal infection was observed in 9 patients (39.13%). Sleep disorder was most common (69.57%), followed by psychiatric disorder and cognitive impairment (65.22%), consciousness disorder (60.87%), seizures (56.52%), speech disorder (39.13%), and headache (39.13%). Radiologically, 5 patients (21.74%) had damage to the cerebral cortex and basal ganglia. 4 patients (17.39%) had damage to the brainstem. All patients received first-line immunotherapy, and 16 (69.57%) of these patients received second-line treatment and long-term immunotherapy. Across 23 clinical attacks, the median modified Rankin Scale score (mRS) improved from 3 [interquartile range (IQR): 3,4] pre-treatment to 1 (IQR: 0,1) post-treatment. Relapses occurred in 6 patients (26.09%). We selected 51 adult patients with anti-NMDARE and 30 adult patients with MOGAD who had onset during the same period as the control groups. In terms of gender, psychiatric disorder, and brainstem involvement, there were significant differences between the anti-NMDARE group and the MNOS group. Regarding visual impairment, limb weakness, deep white matter lesion, and QAlb, differences were observed between the MOGAD group and the MNOS group.

**Discussion:**

When atypical clinical symptoms and imaging results appear, clinicians should consider the possibility of antibody overlap. During anti-NMDARE relapse, attention should be paid to whether anti-MOG antibodies are co-present.

## 1 Introduction

Anti-NMDARE is a severe autoimmune disorder characterized by cerebrospinal fluid (CSF) antibodies against the NR1 subunit of the NMDA receptor, primarily affecting young females. The main clinical features include cognitive dysfunction, psychiatric disorders, dyskinesia, seizures, decreased consciousness, language impairment, autonomic dysfunction, and central hypoventilation caused by cortical involvement. The condition is often associated with ovarian teratomas (1). Approximately 35% of patients exhibit mild and non-specific radiological abnormalities on brain magnetic resonance imaging (MRI) during the acute stage (2). MOG is a surface protein on myelin sheaths in the central nervous system (CNS). Recent evidence has demonstrated that antibodies against MOG are associated with various idiopathic inflammatory demyelinating diseases, collectively termed MOGAD (3). The coexistence of multiple anti-neural antibodies refers to the simultaneous or sequential presence of different autoantibodies within the same patient (4). Several cases have reported the coexistence of NMDAR-ab and MOG-ab which some authors describe as MNOS (5). The clinical features, management, and prognosis of MNOS differ significantly from those of MOGAD or anti-NMDARE, presenting diagnostic and therapeutic challenges. Despite sporadic reports of overlapping NMDAR-ab and MOG-ab, comprehensive knowledge of MNOS remains limited. This study aimed to analyze the clinical features and outcomes of patients with MNOS, further characterizing its features and exploring potential mechanisms underlying the occurrence of dual-positive antibodies.

## 2 Materials and methods

### 2.1 Patients

This was a retrospective study involving adult patients with MNOS from January 2020 to December 2024 in the Affiliated Brain Hospital of Nanjing Medical University. The inclusion criteria were the following: (1) age≥18 years old; (2) diagnosed with anti-NMDARE (6)or MOGAD (7); (3) seropositivity or CSF positivity for MOG-ab; (4) seropositivity or CSF positivity for NMDAR-ab. The exclusion criteria included:(1). positivity for more than 2 specific antibodies against neurons or glial cells;(2) lost to follow up. The inclusion criteria of the control groups were the following: (1) age≥18 years old; (2)diagnosed with anti-NMDARE (6) or MOGAD (7); (3) seropositivity or CSF positivity for only one of the neuronal antibodies (MOG or NMDAR).The exclusion criteria included: (1) seropositivity or CSF positivity for other neuronal antibodies; (2) having a concomitant tumor; (3) lost to follow up. The study was approved by the Ethics Committee of the Affiliated Brain Hospital of Nanjing Medical University. Written informed consent was obtained from all participants prior to inclusion in the study.

### 2.2 Collection of clinical data

We collected demographics, clinical presentations, laboratory data, imaging findings, treatments, and prognoses for all patients. Each patient was followed up for a minimum of 6 months after discharge. The presence of CNS demyelinating antibodies [Aquaporin– 4(AQP4), MOG, myelin basic protein (MBP), or glial fibrillary acidic protein (GFAP)] and autoimmune encephalitis (AE) antibodies [NMDAR, α-amino-3-hydroxy-5-methyl-4-isoxazolepropionic acid receptor subunit 1/2 (AMPA1/2), Leucine-rich glioma-inactivated 1 protein (LGI1), Contactin-associated protein-like 2 (CASPR2), γ-aminobutyric acid type B receptor (GABABR)] was assessed in all patients. MOG-ab was confirmed by live cell-based assay (CBA) testing. NMDAR-ab was confirmed by fixed CBA testing in CSF. Only when the NMDAR-ab in serum was CBA-positive would the specimen be retested positive via tissue immunohistochemistry. Antibody titers in serum and CSF samples were measured by Euroimmun CBA. Serum NMDAR-ab titers ≥1:10 and CSF titers ≥1:1 were defined as positive. Similarly, serum MOG-ab titers ≥1:10 and CSF titers ≥1:1 were defined as positive. The titer was converted to its denominator, and the logarithm of the denominator was calculated for comparison of data differences. The mRS was used to assess neurological disability both at the time of maximum disease severity (MAXmRS) and at discharge (mRS). The MAXmRS-mRS was employed to evaluate the therapeutic effects of first-line immunotherapy. MAXmRS-mRS ≥2 is defined as a favorable therapeutic effect. Relapse was defined as the appearance of new or worsening symptoms lasting at least 24 hours, supported by clinical, laboratory, or imaging findings, and occurring at least 2 months after stabilization or improvement in NMDARE (2, 6) or at least 1 month in MOGAD (8). Therefore, we defined relapse in patients with MNOS as the new onset of anti-NMDARE or MOGAD, which occurred at least one month after the initial stabilization or improvement of first episode of double antibody positivity. Brain magnetic resonance imaging (MRI) was performed using a 3.0 T scanner for all patients. MRI sequences analyzed included T1-weighted, T2-weighted, diffusion-weighted imaging (DWI) and Gd enhancement.

### 2.3 Statistical analysis

Statistical data were analyzed using IBM SPSS Statistics 27 software. Continuous variables following a normal distribution were reported as mean ± standard deviation (SD), whereas those with a skewed distribution were described as median and IQR. Discontinuous variables were presented as frequencies and constituent ratios. For the statistical analysis of intergroup differences, independent samples t-test was used to compare normally distributed continuous variables, Mann–Whitney U test was applied to compare non-normally distributed continuous variables, and Pearson’s chi-square test was employed to compare non-continuous variables. Statistical significance was determined at a threshold of P < 0.05. All data were subjected to the false discovery rate (FDR) test.

## 3 Result

### 3.1 Demographics and clinical features

**Figure 1:**
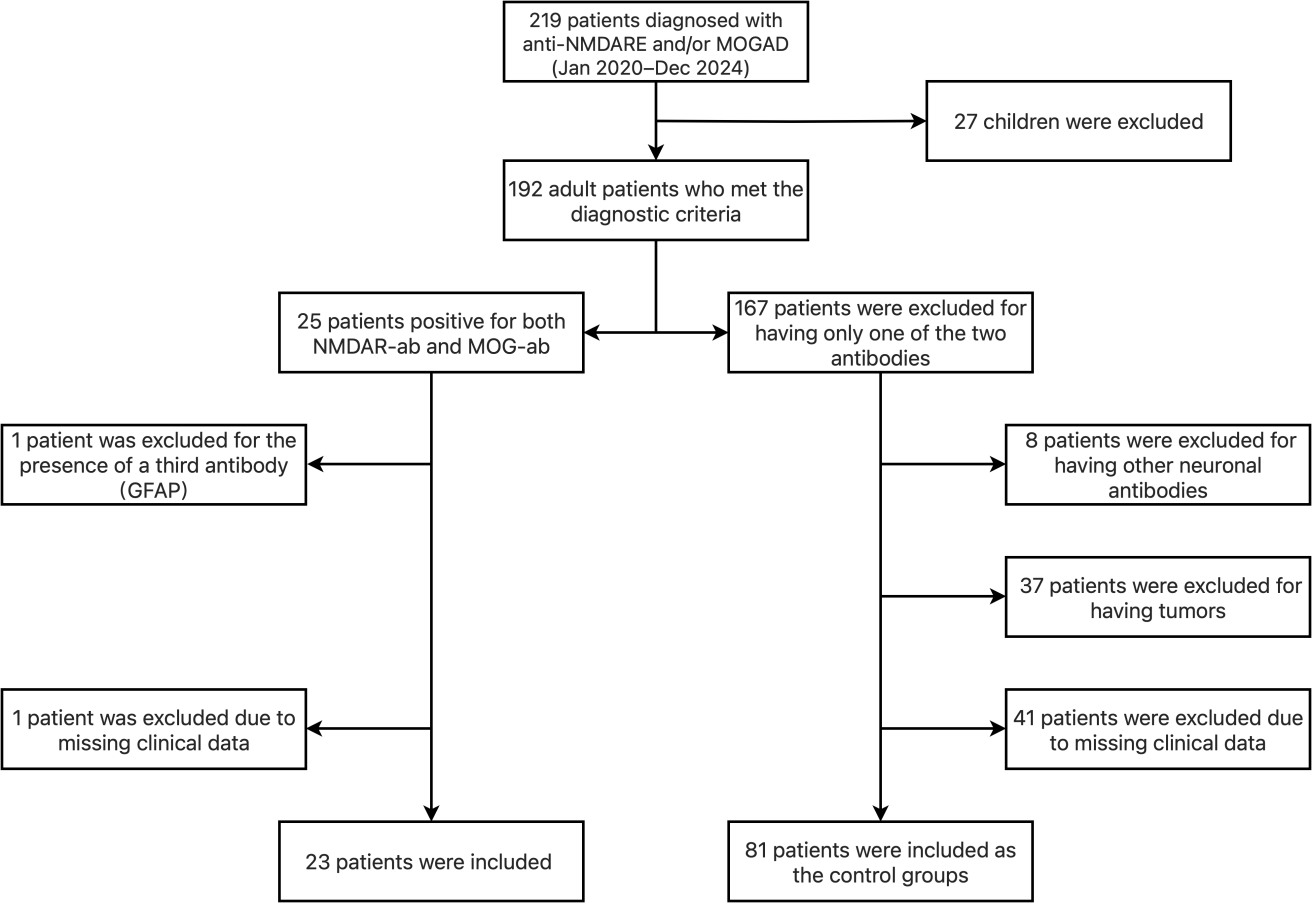
Study flow diagram.

We identified 23 adult patients with overlapping syndrome from 219 patients diagnosed with anti-NMDARE and/or MOGAD at the Affiliated Brain Hospital of Nanjing Medical University (a single-center study) from January 2020 to December 2024. Additionally, we selected 51 adult patients with anti-NMDARE and 30 adult patients with MOGAD who had onset during the same period as the control groups. As shown in **Figure 1**. Among the 23 patients, 6 (26.09%) had a history of monospecific antibody disease before the onset of MNOS, 3 (13.04%) were diagnosed with anti-NMDARE, and 3 (13.04%) were diagnosed with MOGAD. 17 (73.91%) patients were found to have NMDAR-ab and MOG-ab simultaneously at the time of their first onset. Clinical and paraclinical information can be found in detail in **Tables 1**, **2**, and **3** in the **Supplementary Materials**. We analyzed the clinical characteristics of 23 patients with MNOS, comprising 20 males (86.96%) and 3 females (13.04%). The mean age of onset was 36.17 ± 11.21 years old. Among the 23 patients, 10 (43.48%) met the criteria for anti-NMDARE only, 5 (21.74%) met the criteria for MOGAD only, and 8 (34.78%) met the diagnostic criteria for both anti-NMDARE and MOGAD. Among the 13 patients diagnosed with MOGAD, 6 (6/13,46.15%) presented with cortical encephalitis, 2 (2/13,15.38%) with optic neuritis, 2 (2/13,15.38%) with acute disseminated encephalomyelitis (ADEM), 1 (1/13,7.69%) with brainstem encephalitis, 1 (1/13,7.69%) with encephalitis, and 1 (1/13,7.69%) with concurrent optic neuritis and ADEM.9 patients (39.13%) had prodromal infections. No tumors or autoimmune diseases were detected in any patient. The most common symptoms in these patients were sleep disorder(16/23,69.57%),of which 11 (11/16,68.75%) presented with insomnia and 5 (5/16,31.25%) with hypersomnia, psychiatric disorder(15/23, 65.22%), cognitive impairment(15/23, 65.22%), followed by consciousness disorder(14/23, 60.87%), seizures(13/23, 56.52%), speech disorder(9/23,39.13%), headache(9/23,39.13%), limb paralysis(6/23, 26.09%),sphincter disorder (4/23, 17.39%), dyskinesia (4/23,17.39%),ataxia (3/23, 13.04%),visual impairment(3/23, 13.04%) and limb numbness(2/23, 8.70%).

### 3.2 Laboratory features

3 patients (3/23, 13.04%) had increased intracranial pressure (>180mmH2O) with a mean of 153.83 ± 102.10mmH2O, 15(15/23, 65.22%) patients had CSF pleocytosis (>8 white cell count/μL) with a mean of 44.74 ± 75.69 cells/μL and 17 patients(17/23, 73.91%) had increased protein (>0.4g/L) with a mean of 0.51 ± 0.17g/L. 3 patients (3/23,13.04%) had increased QAlb (>9) with a median of 5.97 (range, 1.72-11.83).

### 3.3 Radiological features

15 patients (65.22%) had negative imaging findings.5 patients (21.74%) had cortical involvement,5 patients (21.74%) had basal ganglia involvement,4 (17.39%) patients had brainstem involvement,2 patients (8.70%) showed deep white matter involvement,2 patients (8.70%) had thalamic involvement,2 patients (8.70%) had cerebellar involvement and 1 (4.35%) showed periventricular involvement.

### 3.4 Treatment outcomes and recurrence

All patients received an intravenous high dose of methylprednisolone (HIMP) during the acute phase, 4 (17.39%) patients were concurrently administered intravenous immunoglobulin (IVIG) therapy. 16 (69.57%) patients received second-line treatment and long-term immunotherapy, of whom 14 (14/16,87.50%) were treated with mycophenolate mofetil, 1 (1/14,6.25%) with azathioprine, and 1 (1/14,6.25%) with rituximab. 7 (30.43%) patients did not receive second-line treatment. For 23 clinical attacks, the median mRS before treatment was 3 (IQR: 3,4), decreasing to 1 (IQR: 0,1) after acute-phase treatment. The median MAXmRS-mRS was 3 (IQR:2,3). All patients with MNOS had an MAXmRS-mRS ≥ 2, indicating a significant response to first-line treatment. During the median 25-month follow-up (range: 6–59 months), the mRS scores of all patients were ≤ 2. 6 patients (26.09%) had relapses, the time to recurrence of the 6 patients ranged from 2 to 48 months, with a median of 11 months. Among the relapses, 5 patients (5/6,83.33%) exhibited symptoms more consistent with MOGAD. Only 1 patient (1/6,16.67%) exhibited symptoms more consistent with NMDARE.

### 3.5 Comparison with anti-NMDARE and MOGAD

There were significant differences between the MNOS group and the anti-NMDARE group in terms of gender, psychiatric disorders, and brainstem involvement on MRI. Compared with the anti-NMDARE group, the MNOS group had a higher proportion of males and a higher rate of brainstem involvement on MRI, while the number of patients with psychiatric symptoms was lower.

There were significant differences between the MNOS group and the MOGAD group in terms of gender, psychiatric disorders, disturbances of consciousness, cognitive impairment, visual impairment, sleep disorders, limb weakness, and QAlb. The proportions of patients with psychiatric disorders, disturbances of consciousness, cognitive impairment, and sleep disorders in the MNOS group were higher than those in the MOGAD group. In contrast, the MNOS group had a lower proportion of patients with visual impairment and limb weakness, and a lower QAlb value compared with the MOGAD group. Details are shown in **Table 1**.

**Table 1:** Comparison of the MNOS group with the anti-NMDARE and MOGAD groups.

Characteristics	Anti-NMDARE		MOGAD		MNOS
	N=51	P	N=30	P	N=23
Demographics					
Age	35.16 ± 17.07	0.762	39.50 ± 16.10	0.380	36.17 ± 11.21
Gender	23(45.10%)	0.002	15(50.00%)	0.005	20(86.96%)
Infection	12(23.53%)	0.168	5(16.67%)	0.132	9(39.13%)
Clinical presentation					
Headache	17(33.33%)	0.629	18(60.00%)	0.264	9(39.13%)
Mental abnormality	45(88.24%)	0.019	5(16.67%)	0.002	15(65.22%)
Seizures	26(50.98%)	0.659	9(30.00%)	0.104	13(56.52%)
Conscious disorder	36(70.59%)	0.408	1(3.33%)	0.002	14(60.87%)
Cognitive impairment	39(76.47%)	0.313	6(20.00%)	0.002	15(65.22%)
Sphincter disorder	15(29.41%)	0.273	11(36.67%)	0.246	4(17.39%)
Ataxia	2(3.92%)	0.296	3(10.00%)	0.729	3(13.04%)
Speech disorder	22(43.14%)	0.746	6(20.00%)	0.250	9(39.13%)
Visual impairment	3(5.88%)	0.296	13(43.33%)	0.034	3(13.04%)
Sleep disorder	38(74.51%)	0.658	7(23.33%)	0.002	16(69.57%)
Limb paralysis	11(21.57%)	0.669	18(60.00%)	0.028	6(26.09%)
Limb numbness	7(13.73%)	0.540	7(23.33%)	0.320	2(8.70%)
Dyskinesia	20(39.22%)	0.126	3(10.00%)	0.431	4(17.39%)
Abnormal MRI					
Cortex	15(29.41%)	0.492	14(46.67%)	0.122	5(21.74%)
Deep white matter	5(9.80%)	0.880	10(33.33%)	0.068	2(8.70%)
Periventricular	1(1.96%)	0.558	3(10.00%)	0.558	1(4.35%)
Basal ganglion	9(17.65%)	0.677	6(20.00%)	0.877	5(21.74%)
Thalamus	2(3.92%)	0.401	4(13.33%)	0.597	2(8.70%)
Brainstem	1(1.96%)	0.028	9(30.00%)	0.290	4(17.39%)
Cerebellum	0(0.00%)	0.066	2(6.67%)	0.782	2(8.70%)
CSF					
QAlb	5.67(4.42,8.16)	0.939	7.92(5.72,9.92)	0.044	5.97(4.27,7.14)
pressure	145.00 ± 73.27	0.674	132.07 ± 67.41	0.355	153.83 ± 102.10
leukocyte	35.47 ± 53.39	0.548	95.40 ± 152.78	0.121	44.74 ± 75.69
protein	0.51 ± 0.26	0.945	0.66 ± 0.37	0.050	0.51 ± 0.17
Positive ab					
NMDAR-ab serum	1(-0.30,1.51)	0.683	–	–	1(-0.30,1.51)
NMDAR-ab CSF	1(0.00,1.51)	0.347	–	–	0.51(0.00,1.51)
MOG-ab serum	–	–	1.51(1.00,2.00)	0.640	1.51(0.51,2.00)
MOG-ab CSF	–	–	0(-0.30,1.00)	0.715	0.51(-0.30,1.00)
Relapse	9(17.65%)	0.403	9(30.00%)	0.754	6(26.09%)
Scale scores					
MAXmRS	3(3,5)	0.685	3(3,4)	0.306	3(3,4)
mRS	1(0,2)	0.127	0(0,1)	0.622	1(0,1)
MAXmRS-mRS	2(2,3)	0.166	3(2,3)	0.183	3(2,3)

## 4 Discussion

The coexistence of autoimmune antibodies has captured the attention of researchers. This is due to certain atypical symptoms or lesions that cannot be accounted for by a single antibody. Among these, the overlap of NMDAR-ab and MOG-ab is more frequently observed in clinical practice (6, 9).As early as in the report by Titulaer, out of 691 patients diagnosed with anti-NMDARE, 12 patients exhibited MRI or clinical manifestations of demyelination, and these 12 patients were detected to be positive for MOG-ab (10). With the widespread application of antibody detection techniques, the number of relevant studies has been on the increase. However, reports on adult patients with the MNOS remain scarce, and their clinical features are not yet clearly elaborated. Therefore, this study aimed to describe the clinical features, the response to first-line treatment, and the recurrence situation of 23 adult patients with the MNOS. Furthermore, this was compared with the anti-NMDARE and MOGAD control groups to facilitate differential diagnosis and precision medicine in clinical practice.

Our patients showed a male predominance and most of them were young and middle-aged, which is in accordance with the formal reports (11, 12). In this study, the proportion of males in the MNOS group was significantly higher than that in the anti-NMDARE group and the MOGAD group, consistent with previous findings (13). Because MOGAD shows a relatively balanced gender distribution with a slight male predominance (3), we deduced that the gender distribution of MNOS may be influenced by that of MOG-ab. Therefore, MNOS should be more vigilantly considered in middle-aged male patients with anti-NMDARE. Consistent with previous studies, no tumours were found to be concurrent in any of the patients (10). Given that many patients with anti-NMDARE suffer from ovarian teratoma (2, 13), this indicated that MNOS has a pathogenic mechanism different from that of encephalitis (tumour–related). Non-paraneoplastic patients are prone to having overlapping antibodies (10). Additionally, the non-tumorigenic onset mechanism of MNOS, distinct from anti-NMDARE, may contribute to the lower incidence in females. Our study found 9 out of 23 patients had prodromal infection, revealing that infection may be the disease triggers, consistent with the formal reports (10, 15).

We found that sleep disorder was a relatively common symptom among MNOS patients, followed by psychiatric disorders, consciousness and cognitive impairment, and epilepsy. In other words, most patients exhibited encephalitic symptoms during their initial episode. In line with past studies, the clinical manifestations of patients with MNOS are predominantly characterized by anti-NMDARE (10, 16). However, these patients presenting with anti-NMDARE may also present with some atypical symptoms, including limb paralysis, sphincter disorder, ataxia, visual impairment and limb numbness, which are symptoms of MOGAD. Therefore, by observing these atypical clinical symptoms in anti-NMDARE, valuable clues regarding the coexistence of antibodies can be obtained. In our study, MNOS patients had fewer psychiatric symptoms than the anti-NMDARE group but more than the MOGAD group, consistent with previous studies (9, 13, 17). This suggests that the possibility of antibody coexistence should be fully considered in anti-NMDARE patients without psychiatric symptoms or MOGAD patients with psychiatric symptoms. The proportions of visual impairment and limb weakness in MNOS were lower than those in MOGAD, while the rates of disturbances of consciousness, cognitive impairment, and sleep disorders were higher, which is in line with previous findings (13, 17, 18). Accordingly, MNOS should be considered in MOGAD patients who lack visual impairment or limb weakness but present with disturbances of consciousness, cognitive impairment, or sleep disorders. In our study, the BBB was more susceptible to disruption in MOGAD than in the MNOS group, possibly due to the greater propensity of MOG-ab to damage the BBB (19).

**Figure 2:**
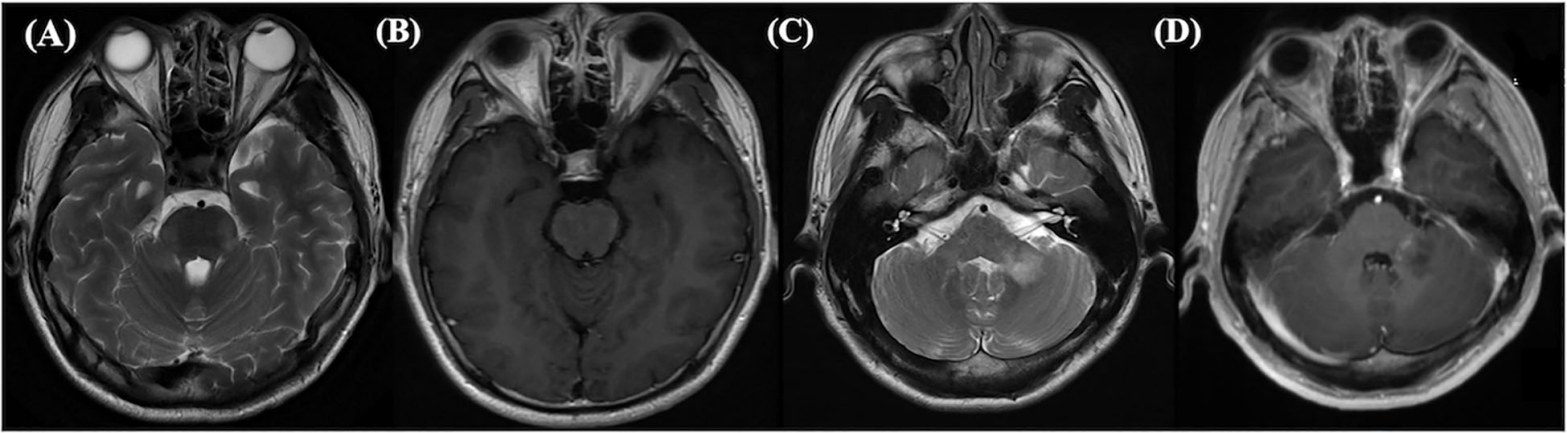
This abnormal MRI was from a 28-year-old male suffered from NMDARE then transferred to MOGAD. Axial T2-weighted **(A)** and enhancement **(B)** images showed hyperintense signals in brain pons, bilateral middle cerebellar peduncle and medulla oblongata at the first relapse. At the second relapse, left cerebellopontine area showed hypersignals in T2-weighted **(C)** and enhancement **(D)**. Both relapses were double antibody positive.

**Figure 3:**
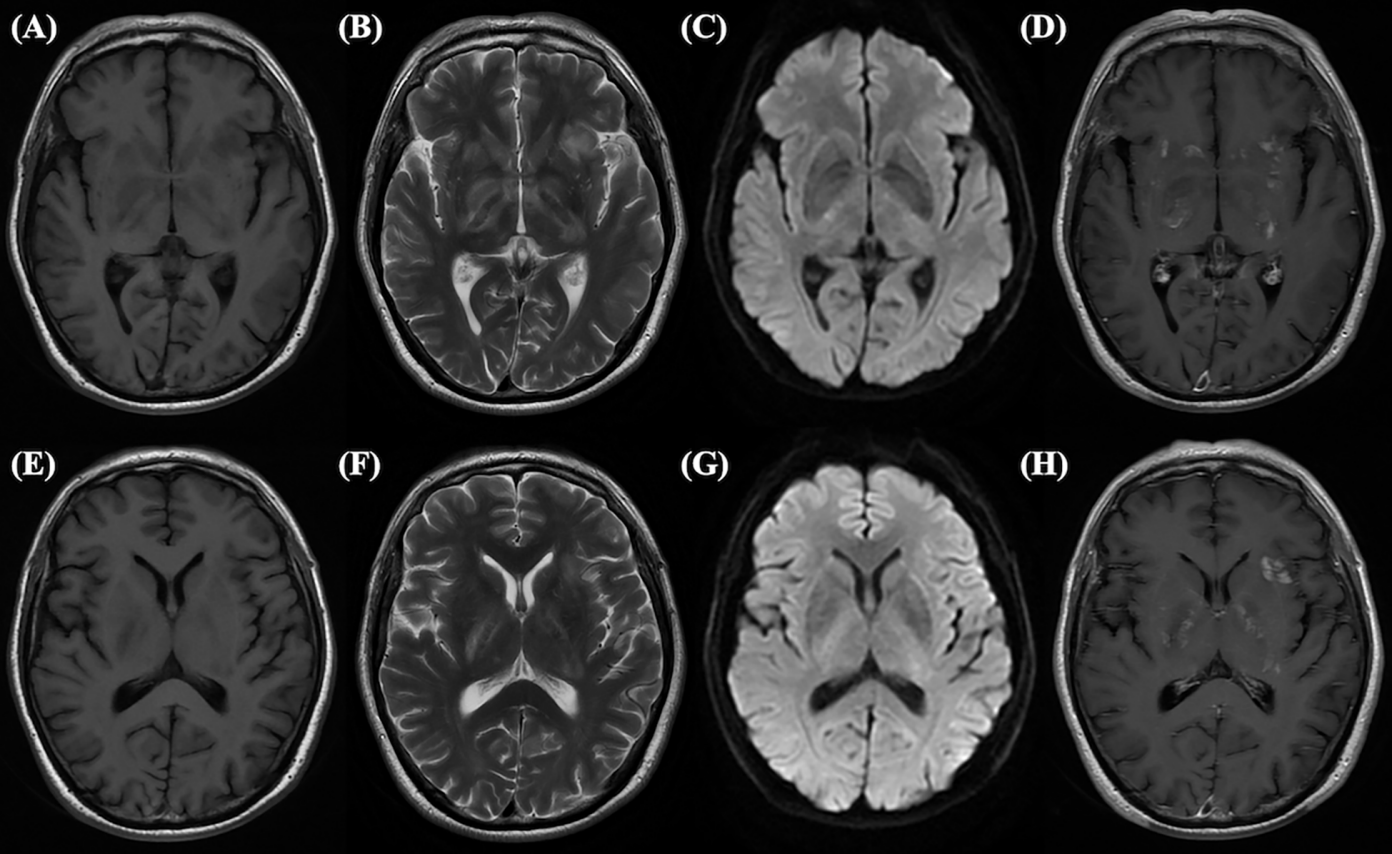
This abnormal MRI is from a 65-year-old male, who tested positive for double antibodies upon first admission. Long signals on T1-weighted **(A)** and T2-weighted **(B)** images, slightly high signals on DWI **(C)**, and enhanced shadows on Gd enhancement **(D)** can be seen in the hippocampus. Long signals on T1-weighted **(E)** and T2-weighted **(F)** images, slightly high signals on DWI **(G)**, and enhanced shadows on Gd enhancement **(H)** can be seen in the basal ganglion.

In our study, patients with positive MRI findings exhibited overlapping manifestations of MOGAD and anti-NMDARE, such as involvement of the cerebral cortex, basal ganglia, and brainstem, (**Figures 2**, **3**), which is consistent with previous studies (5). Patients with anti-NMDARE typically show normal or mildly abnormal MRI findings, presenting as scattered cortical or subcortical T2-weighted hyperintensities, with a few cases showing inflammatory demyelinating changes involving the brainstem and white matter (14). In contrast, MOGAD involved more extensive imaging abnormalities, affecting the cortical grey matter, subcortical white matter, deep white matter, brainstem, basal ganglia, thalamus, and cerebellum, presenting as poorly demarcated hyperintensities on T2-weighted images (20, 21).It has been suggested that when anti-NMDARE patients present with positive imaging findings, especially demyelinating changes in the white matter and infratentorial regions, the presence of MNOS should be considered. The MNOS group showed a higher rate of brainstem involvement on MRI than the anti-NMDARE group. Previous research indicated that the brainstem is the most frequently involved brain structure in MNOS patients (5).

Currently, there is no unified consensus regarding the clinical treatment of MNOS. The principal therapeutic approaches are mainly founded on the existing treatments for NMDARE and MOGAD. Our patients were treated in accordance with the treatment principles for both diseases (22, 23), and all achieved favorable therapeutic effects. This indicates that the existing treatment principles have demonstrated a favourable therapeutic effect on patients in the acute phase, as reported in previous studies (12, 24).Additionally, we observed that most patients exhibited a favorable response to first-line treatment within two weeks; consequently, second-line treatment was less frequently used in our patient cohort (23).In our cohort, 6 (26.09%) of our patients experienced relapses. In contrast, other studies reported higher recurrence rates. Specifically, one study had a recurrence rate of 64% (16), and a meta-analysis indicated that the recurrence rate in patients with MNOS reached 63.4% (18). Among the relapses, 5 patients (5/6,83.33%) exhibited symptoms more consistent with MOGAD.From this, it can be deduced that when MOG-ab acts as a pathogenic antibody, the relapse rate is likely to increase. Moreover, MOG-ab was more likely to coexist with NMDAR-ab in relapsed patients (9).

Our study has limitations. First, the sample size was small as it was from only one affiliated hospital of a medical university, limiting the generalizability of results. Second, the short follow-up period might overestimate glucocorticoid efficacy and underestimate the disease recurrence rate, failing to fully capture long-term treatment effects and recurrence patterns.

## 5 Conclusion

When patients diagnosed MOGAD or anti-NMDARE presented with clinical manifestations that deviate from the typical symptom profiles, such as unusual neurological deficits, atypical progression patterns, or overlapping features, clinicians should maintain a high index of suspicion for antibody overlap. Notably, during the relapse phase of anti-NMDARE, special attention should be directed toward reevaluating the presence of MOG-ab. Relapses in anti-NMDARE may occasionally coincide with the emergence or reactivation of MOG-ab.

## Data Availability

The original contributions presented in the study are included in the article/**Supplementary Material**. Further inquiries can be directed to the corresponding author.
