# Supplementary material for: Clinical features and prognosis of the adult patients positive for both N-methyl-d-aspartate receptor and myelin oligodendrocyte glycoprotein antibodies
Source: Front Immunol. 2025 Oct 16;16:1664666. doi: 10.3389/fimmu.2025.1664666 (PMC12571725; doi:10.3389/fimmu.2025.1664666)
Supplement: Supplementary file 1 [file Table1.docx]

Table1 Clinical features of anti-NMDARE episodes (A) and MNOS episodes (B) in 3 patients with temporally separated occurrences of these two disorders.

1. Episodes of anti-NMDARE

| No | Age/  Gender | Interval/M | Clinical presentation | MRI | CSF WC / Protein | NMDAR-ab | Therapy | MAXmRS/  mRS |
| --- | --- | --- | --- | --- | --- | --- | --- | --- |
| 1 | 25/M | 41 | Mental abnormality, Conscious disorder, Cognitive impairment, Seizures, Sphincter disorder, Hypersomnia, Dyskinesia | - | 53/0.34 | CSF: 1:30  Serum: - | St, IVIG，MMF | 5/2 |
| 2 | 36/M | 92 | Headache, Seizures, Dyskinesia, Speech disorder | Cortex | 130/0.53 | CSF: 1:1  Serum: - | St, IVIG | 3/1 |
| 3 | 48/F | 24 | Mental abnormality, Conscious disorder, Cognitive impairment，Insomnia, | - | Na | CSF: 1:10  Serum: - | St, IVIG | 4/2 |

(B) Episodes of MNOS

| No | Age | Diagnosis | Clinical presentation/MOGAD classification | MRI | CSF WC / Protein | NMDAR-ab | MOG-ab | Therapy | Relapse/M | Relapse symptoms | MAXmRS/  mRS |
| --- | --- | --- | --- | --- | --- | --- | --- | --- | --- | --- | --- |
| 1 | 28 | MOGAD | Ataxia/ Brainstem encephalitis | Brainstem, Cerebellum | 7/0.57 | CSF: 1:10  Serum: 1:10 | Serum: 1:100 | St，MMF | +/16 | Ataxia | 3/0 |
| 2 | 43 | Anti-NMDARE+MOGAD | Headache, Seizures, Conscious disorder, Insomnia, Dyskinesia/ Cortical encephalitis | Cortex | 35/0.4 | CSF: 1:3.2  Serum: 1:10 | CSF: 1:3.2  Serum: 1:100 | St, IVIG, MMF | +/2 | Mental abnormality, sleep disorder | 4/1 |
| 3 | 50 | Anti-NMDARE+MOGAD | Mental abnormality, Seizures, Conscious disorder  Cognitive impairment, Sphincter disorder, Hypersomnia / Cortical encephalitis | Cortex, Basal ganglion, Thalamus, Brainstem, Cerebellum | 3/0.41 | CSF: 1:10  Serum: 1:32 | CSF: 1:32  Serum: 1:100 | St, MMF | +/36 | Mental abnormality, Speech disorder | 3/1 |

M, male; F, female; M, month; MRI, Brain magnetic resonance imaging; anti-NMDARE, anti- N-methyl-d-aspartate receptor encephalitis; MOGAD, myelin oligodendrocyte glycoprotein antibody-associated disorders; WC, white blood cells; ab, antibodies; CSF, cerebrospinal fluid; St, steroids; IVIG, intravenous immunoglobulins; MMF, mycophenolate mofetil; mRS, modified Rankin Scale; max, maximum; Na, not applicable.

Antibody tests were all conducted during the symptomatic phase of the disease and prior to first-line treatment.

Table2 Clinical features of MOGAD episodes (A) and MNOS episodes (B) in 3 patients with temporally separated occurrences of these two disorders.

(A) Episodes of MOGAD

| No | Age/  Gender | Interval/M | Clinical presentation/MOGAD classification | MRI | CSF WC / Protein | MOG-ab | Therapy | MAXmRS/  mRS |
| --- | --- | --- | --- | --- | --- | --- | --- | --- |
| 1 | 32/M | 11 | Headache, Seizures/ Cortical encephalitis | Cortex | 78/0.51 | CSF: 1:32  Serum: - | St | 3/1 |
| 2 | 37/M | 14 | Visual impairment/ Optic neuritis | - | Nd | CSF: -  Serum: 1:100 | St | 3/1 |
| 3 | 30/M | 33 | Visual impairment, Ataxia/ Optic neuritis, Adem | Cortex, Deep white matter, Basal ganglion, Brainstem | Na | CSF: -  Serum: 1:32 | St | 4/1 |

(B) Episodes of MNOS

| No | Age | Diagnosis | Clinical presentation/MOGAD classification | MRI | CSF WC / Protein | NMDAR-ab | MOG-ab | Therapy | Relapse/M | Relapse symptoms | MAXmRS/  mRS |
| --- | --- | --- | --- | --- | --- | --- | --- | --- | --- | --- | --- |
| 1 | 33 | MOGAD | Headache, Seizures/ Cortical encephalitis | - | 20/0.40 | CSF: 1:1  Serum: - | CSF: 1:3.2  Serum: 1:100 | St, MMF | - | - | 3/0 |
| 2 | 39 | Anti-NMDARE+MOGAD | Mental abnormality, Seizures, Conscious disorder,  Cognitive impairment, Speech disorder, Visual impairment, insomnia/ Optic neuritis | - | 81/0.46 | CSF: 1:100  Serum: 1:100 | CSF: 1:10  Serum: - | St, IVIG, MMF | - | - | 4/0 |
| 3 | 33 | Anti-NMDARE+MOGAD | Mental abnormality,  Conscious disorder,  Cognitive impairment,  Ataxia,  Visual impairment,  Limb paralysis, Limb numbness/ Optic neuritis, Adem | Cortex,  Deep white matter  Basal ganglion, Brainstem | 8/0.43 | CSF: -  Serum: 1:100 | CSF: -  Serum: 1:100 | St, AZA | +/4 | Visual impairment,  Limb paralysis, Limb numbness | 4/2 |

M, male; M, month; MRI, Brain magnetic resonance imaging; anti-NMDARE, anti- N-methyl-d-aspartate receptor encephalitis; MOGAD, myelin oligodendrocyte glycoprotein antibody-associated disorders; WC, white blood cells; ab, antibodies; CSF, cerebrospinal fluid; St, steroids; IVIG, intravenous immunoglobulins; MMF, mycophenolate mofetil; AZA, azathioprine; mRS, modified Rankin Scale; max, maximum; Nd, not done.

Antibody tests were all conducted during the symptomatic phase of the disease and prior to first-line treatment.

Table3 Clinical features of 17 patients with MNOS at the time of their first onset.

| No | Age/Gender | Diagnosis | Clinical presentation/MOGAD classification | MRI | CSF WC / Protein | NMDAR-ab | MOG-ab | Therapy | Relapse/M | Relapse symptoms | MAXmRS/  mRS |
| --- | --- | --- | --- | --- | --- | --- | --- | --- | --- | --- | --- |
| 1 | 26/M | Anti-NMDARE | Headache, Mental abnormality, Conscious disorder,  Cognitive impairment, Speech disorder, insomnia, Limb paralysis/- | - | 25/0.23 | CSF: 1:1  Serum: 1:10 | CSF: 1:1  Serum: 1:32 | St, IVIG | - | - | 4/2 |
| 2 | 50/M | Anti-NMDARE | Mental abnormality, Seizures, Conscious disorder, insomnia/- | - | 16/0.41 | CSF: 1:32  Serum: 1:10 | CSF: 1:10  Serum: - | St,MMF | - | - | 3/0 |
| 3 | 20/M | Anti-NMDARE | Headache, Mental abnormality, Seizures, Conscious disorder,  Cognitive impairment, insomnia/- | - | 2/0.44 | CSF: 1:100  Serum: 1:100 | CSF: -  Serum: 1:32 | St | - | - | 3/1 |
| 4 | 43/M | Anti-NMDARE | Mental abnormality, Seizures, Conscious disorder,  Cognitive impairment, Sphincter disorder, insomnia/- | - | 7/0.31 | CSF: -  Serum: 1:10 | CSF: -  Serum: 1:32 | St | - | - | 4/1 |
| 5 | 26/M | Anti-NMDARE | Mental abnormality, Seizures, Cognitive impairment, Speech disorder/- | - | 34/0.50 | CSF: 1:3.2  Serum: 1:10 | CSF: 1:3.2  Serum: 1:3.2 | St,MMF | - | - | 3/1 |
| 6 | 30/M | Anti-NMDARE | Mental abnormality, Conscious disorder, Cognitive impairment, Speech disorder, Hypersomnia, Limb paralysis, Dyskinesia/- | - | 23/0.50 | CSF: 1:3.2  Serum: 1:32 | CSF: -  Serum: 1:10 | St | - | - | 4/1 |
| 7 | 39/M | Anti-NMDARE | Mental abnormality, Seizures, Conscious disorder, Cognitive impairment, Hypersomnia/- | - | 50/0.57 | CSF: 1:3.2  Serum: 1:3.2 | CSF: 1:3.2  Serum: 1:3.2 | St | - | - | 4/1 |
| 8 | 39/M | Anti-NMDARE | Headache, Mental abnormality, Conscious disorder, Cognitive impairment, insomnia, Dyskinesia/- | - | 20/0.60 | CSF: 1:100  Serum: 1:10 | CSF: 1:10  Serum: - | St,MMF | - | - | 3/0 |
| 9 | 28/M | Anti-NMDARE | Headache, Mental abnormality, Conscious disorder, Cognitive impairment, Speech disorder, Hypersomnia/- | - | 333/0.94 | CSF: 1:10  Serum: - | CSF: 1:3.2  Serum: - | St | - | - | 3/1 |
| 10 | 30/M | Anti-NMDARE | Mental abnormality, Seizures, Conscious disorder, Cognitive impairment, Sphincter disorder, insomnia/- | - | 13/0.54 | CSF: 1:100  Serum: - | CSF: 1:10  Serum: - | St, IVIG,RTX | +/48 | Mental abnormality, Seizures, | 5/1 |
| 11 | 26/F | MOGAD | Headache, Visual impairment, Hypersomnia/ On | - | 159/0.44 | CSF: 1:1  Serum: - | CSF: 1:1  Serum: 1:100 | St,MMF | +/6 | Visual impairment, Limb numbness | 3/1 |
| 12 | 23/M | MOGAD | Speech disorder, Limb paralysis/Adem | Deep white matter, Basal ganglion, | 15/0.54 | CSF: 1:32  Serum: - | CSF: -  Serum: 1:32 | St,MMF | - | - | 2/0 |
| 13 | 33/M | MOGAD | Headache, insomnia/ encephalitis | Periventricular, Basal ganglion | 146/0.59 | CSF: 1:3.2  Serum: 1:3.2 | CSF: -  Serum: 1:1000 | St,MMF | - | - | 2/0 |
| 14 | 38/M | Anti-NMDARE+MOGAD | Mental abnormality, Seizures, Conscious disorder, Cognitive impairment, Sphincter disorder, Ataxia, Speech disorder, insomnia, Dyskinesia/ cortical encephalitis | - | 1/0.30 | CSF: 1:10  Serum: 1:32 | CSF: 1:1  Serum: 1:100 | St,MMF | - | - | 5/2 |
| 15 | 33/F | Anti-NMDARE+MOGAD | Headache, Seizures, insomnia/ cortical encephalitis | - | 8/0.38 | CSF: 1:1  Serum: - | CSF: 1:3.2  Serum: 1:10 | St | - | - | 3/0 |
| 16 | 57/M | Anti-NMDARE+MOGAD | Seizures, Cognitive impairment, Speech disorder, Limb paralysis, Limb numbness/ cortical encephalitis | Cortex, | 15/0.80 | CSF: 1:1  Serum: - | CSF: 1:10  Serum: 1:320 | St,MMF | - | - | 4/0 |
| 17 | 65/M | Anti-NMDARE+MOGAD | Mental abnormality, Cognitive impairment, Speech disorder, Limb paralysis/Adem | Cortex, Basal ganglion, Thalamus, Brainstem | 8/0.86 | CSF: 1:1  Serum: - | CSF: -  Serum: 1:10 | St,MMF | - | - | 4/0 |

M, male; F, female; MRI, Brain magnetic resonance imaging; anti-NMDARE, anti- N-methyl-d-aspartate receptor encephalitis; MOGAD, myelin oligodendrocyte glycoprotein antibody-associated disorders; WC, white blood cells; ab, antibodies; CSF, cerebrospinal fluid; St, steroids; IVIG, intravenous immunoglobulins; MMF, mycophenolate mofetil; RTX, rituximab; M, month; mRS, modified Rankin Scale; max, maximum.

Antibody tests were all conducted during the symptomatic phase of the disease and prior to first-line treatment.
